# Low-Complexity MUSIC-Based Direction-of-Arrival Detection Algorithm for Frequency-Modulated Continuous-Wave Vital Radar

**DOI:** 10.3390/s20154295

**Published:** 2020-07-31

**Authors:** Bong-seok Kim, Youngseok Jin, Jonghun Lee, Sangdong Kim

**Affiliations:** Advanced Radar Technology Laboratory, Daegu Gyeongbuk Institute of Science & Technology (DGIST), Daegu 42988, Korea; remnant@dgist.ac.kr (B.-s.K.); ysjin@dgist.ac.kr (Y.J.); jhlee@dgist.ac.kr (J.L.)

**Keywords:** FMCW, array spacing, low complexity, MUSIC

## Abstract

This paper proposes a low complexity multiple-signal-classifier (MUSIC)-based direction-of-arrival (DOA) detection algorithm for frequency-modulated continuous-wave (FMCW) vital radars. In order to reduce redundant complexity, the proposed algorithm employs characteristics of distance between adjacent arrays having trade-offs between field of view (FOV) and resolution performance. First, the proposed algorithm performs coarse DOA estimation using fast Fourier transform. On the basis of the coarse DOA estimation, the number of channels as input of the MUSIC algorithm are selected. If the estimated DOA is smaller than 30${}^{{}^{\circ}}$, it implies that there is an FOV margin. Therefore, the proposed algorithm employs only half of the channels, that is, it is the same as doubling the spacing between arrays. By doing so, the proposed algorithm achieves more than 40% complexity reduction compared to the conventional MUSIC algorithm while achieving similar performance. By experiments, it is shown that the proposed algorithm despite the low complexity is enable to distinguish the adjacent DOA in a practical environment.

## 1. Introduction

Recently, several studies on radar sensors have been reported due to their many advantages, such as being robust against environmental effects, for example, dust and light compared to other sensors [1,2,3]. Radar systems are used in a variety of applications such as weather, military, the automotive industry, vital-sign monitoring, and surveillance [4]. Meanwhile, among various approaches using radar sensors, vital-sign applications have attracted much attention because of their non-contact merits [5,6,7].

In Reference [5], the authors discussed state-of-the-art techniques for vital-sign monitoring using an impulse radio ultrawide-band transceiver, and presented the contributions of different authors regarding the vital signs of a stationary human target. In Reference [6], in order to distinguish multiple targets, the authors employed high-resolution algorithms with low complexity. In Reference [8], Shekh M. M. Islam et al. have proposed a hybrid method consisting of an signal to noise (SNR)-based intelligent decision algorithm for phase-comparison monopulse continuous wave (CW) radar in order to overcome the monitoring limits of multiple targets. Due to the use of monopulse CW radar, the complexity of this system is very low. In addition, this algorithm has worked well with high accuracy by employing independent component analysis with the joint approximation diagonalization of Eigen-matrices algorithm, so called, ICA-JADE algorithm and DOA.

Radar systems are widely used to detect range, Doppler, and angle of targets. In Reference [9], authors have developed ultra-wide-band coherent random noise radar operating over 1∼2 GHz frequency band. This system performed phase coherence by employing heterodyne correlation of the received signal with time-delayed frequency shifted replica of transmit waveform. They have ensured that the reflected signal, when mixed with timed-delayed transmit signal, yields the same intermediate frequency by analysis of theoretical and simulations result. Meanwhile, in Reference [10], Ruosi Sun et al. have considered frame arrival detection issue in the challenging low SNR environment. They have proposed a new detection algorithm by employing the matched filtering and delayed autocorrelation on the filtered signal. In Reference [11], the authors presented that the angle diversity technique implemented on a real-time location system according to the angle estimation, operating in 2.4 GHz band. They theoretically analyzed some problems inherent to the interferometers relative to the noise-level variation and influence of various radio frequency interferences. In Reference [12], they have designed a new distributed angle estimation algorithm in multi-path environment. They have proposed angle estimation algorithm based on received signal strength by using two-antenna anchor. It is shown that the angle is estimated at each sensor in a completely distributed manner.

Among various radar systems, frequency-modulated continuous-wave (FMCW) techniques have attracted much attention as one of the most promising due to their many advantages [13,14,15,16,17,18,19]. In the case of continuous-wave (CW) radar systems, these systems have the merit of low complexity, but they cannot detect the range of targets without additional modulations [1]. In the case of pulsed radar systems, they use very narrow pulse waveforms and, thus, not only are the ranges of targets estimated by using the delay of pulses, but the velocity of targets is also detected by the Doppler effect. These systems require a very large bandwidth at the baseband due to the narrow pulse. Therefore, pulse radar systems require high-cost hardware [2]. Meanwhile, FMCW radar systems overcome drawbacks of CW and pulse radars. Compared to CW radar systems, FMCW radar systems estimate the range of a target as well as its velocity because they employ not only the frequency but also the time domain. Compared to pulse radar systems, an FMCW radar does not require high bandwidth by employing only beat frequency according to delay.

In FMCW radar systems, estimating the direction of arrival (DOA) is a major challenge due to limiting the number of arrays [20,21,22,23]. For DOA estimation, the fast Fourier transform (FFT)-based estimation method is most widely used in FMCW radar due to its low complexity. The FFT-based algorithm has a disadvantage in that it cannot distinguish between adjacent targets due to its low-resolution characteristics. In order to overcome this drawback of the FFT-based algorithm for FMCW radar systems, super-resolution algorithms such as the estimation of signal parameters via rotational invariance technique (ESPRIT) and multiple-signal classifier (MUSIC) were proposed because they have a significantly higher resolution compared with that of the FFT-based algorithm [23,24,25,26,27,28,29,30,31,32]. In References [23,24,25], they tried to detect DOA of adjacent multiple targets by employing MUSIC algorithms. In Reference [26], they attempted to estimate and resolve the overlapping Doppler spectra due to the relatively closer velocities of multiple targets. However, the computational complexity of the super-resolution algorithms is significantly higher compared to FFT-based algorithms and thus super-resolution algorithms are difficult to apply to real-time embedded systems.

In this paper, we propose a low-complexity MUSIC-based DOA detection algorithm for an FMCW vital radar. The proposed algorithm is an extension to the tradeoffs of the conventional MUSIC algorithm. In other words, the proposed algorithm uses the trade-off between the resolution and field of view (FOV) of distance between arrays. As the distance between arrays increases, resolution performance is improved, but the FOV decreases. By employing these characteristics, the proposed algorithm first coarsely estimates the angles of targets by using FFT. Then, the proposed algorithm determines whether the estimated angle of target θ is within $30^{{}^{\circ}}$ or not. In the case of $\theta \leq 30^{{}^{\circ}}$, the proposed algorithm employs only half of the total received (RX) signals as input for the MUSIC algorithms as if the distance between arrays is doubled. In order to increase the signal-to-noise ratio (SNR), the proposed algorithm combines the odd and even signals in the total RX signals. Simulation results showed that the proposed algorithm achieved similar performance to that of the conventional MUSIC algorithm despite the decrease of complexity. In addition, the improvement of SNR by combining odd and even signals was shown by simulations. Experiment results showed the effectiveness of the proposed algorithm in a practical environment.

The remainder of this paper is organized as follows. Section 2 describes the system model considered in this paper and the trade-off between FOV and resolution. Section 3 explains the FMCW radar estimators, that is, the FFT-based and MUSIC algorithms. The proposed low-complexity MUSIC algorithm is introduced in Section 4. In Section 5 and Section 6, the performance and complexity of the proposed algorithm are evaluated through simulations and an experiment. Finally, we present the conclusion in Section 7.

## 2. System Models and Trade-Off between FOV and Resolution

This section illustrates the system models of the vital FMCW radar and the trade-off between FOV and resolution.

### 2.1. System Model

**Figure 1:**
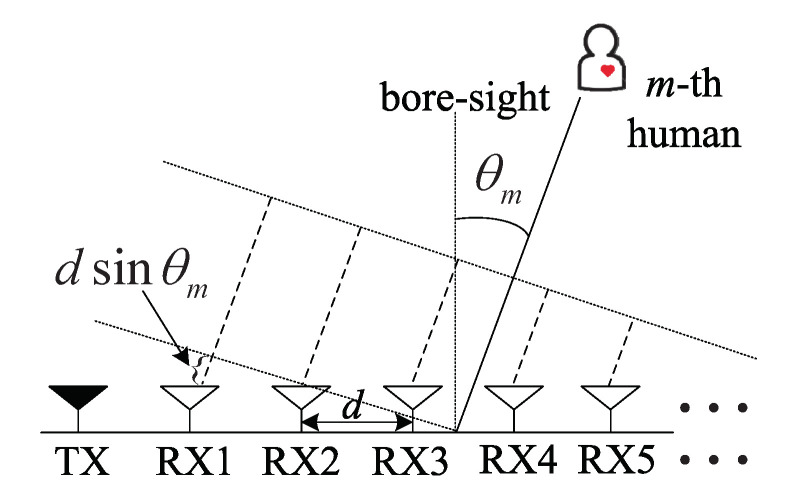
Transmit and receive antennas with unit linear array.

**Figure 2:**
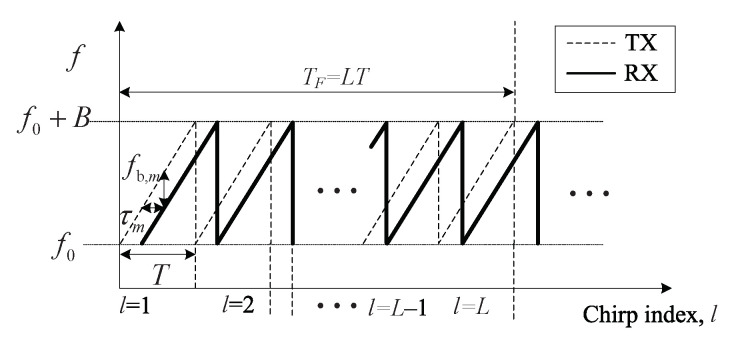
Transmit and receive signals of frequency-modulated continuous-wave (FMCW) radar.

We considered an FMCW radar that was composed on a 1 TX antenna and *K* RX antennas as shown in Figure 1. The FMCW-transmitted (TX) signal $s^{\mathrm{TX}}\left(t\right)$ is composed of *L* chirp signals and expressed as

(1) $$\begin{array}{c} s^{\mathrm{TX}}\left(t\right)=\sum_{l=0}^{L-1}s_{0}\left(t-lT\right), \end{array}$$

where $s_{0}\left(t\right)$ is the chirp signal, that is, $s_{0}\left(t\right)=\exp\left(j\left(2\pi f_{0}t+\mu t^{2}/2\right)\right)$ and $f_{0}$ is the carrier frequency, μ is the chirp slope, that is, μ=B/T, where *B* is the bandwidth and *T* is the symbol duration of a chirp signal as shown in Figure 2.

The TX signal $s^{\mathrm{TX}}\left(t\right)$ is transmitted from the TX antenna and reflected from humans. Then, $s^{\mathrm{TX}}\left(t\right)$ is received at the *k*-th received (RX) antenna and the *l*-th chirp index, and it is denoted by $s_{k,l}^{\mathrm{RX}}\left(t\right)$ and expressed as [6]:

(2) $$\begin{array}{c} s_{k,l}^{\mathrm{RX}}\left(t\right)=\sum_{m=1}^{M}{\tilde{a}}_{m}s^{\mathrm{TX}}\left(t-\tau_{m}\right)\underset{\mathrm{Doppler}\;\mathrm{term}}{\underset{︸}{\exp\left(\frac{j4\pi lT}{\lambda }\left(x_{m}^{\left(\mathrm{hr}\right)}\left(t\right)+\sum_{h=1}^{H}x_{m,h}^{\left(\mathrm{rr}\right)}\left(t\right)\right)\right)}}\underset{\mathrm{DOA}\;\mathrm{term}}{\underset{︸}{\exp\left(\frac{j2\pi }{\lambda }dk\sin\theta_{m}\right)}}+\tilde{w}\left(t\right), \end{array}$$

where ${\tilde{a}}_{m}$ and $\tau_{m}$ are the amplitude and delay of the reflected signal by the *m*-th human, respectively, and $\tilde{w}\left(t\right)$ is the complex additive white Gaussian noise (AWGN). In Doppler terms, $x_{m}^{\left(\mathrm{hr}\right)}\left(t\right)$ denotes the heart rate of the *m*-th human, and $x_{m,h}^{\left(\mathrm{rr}\right)}$ denotes the *h*-th harmonic element of the respiratory rate of the *m*-th human. In DOA terms, *d* is the distance between adjacent RX antennas, λ is the wavelength, $\theta_{m}$ is the DOA term of the *m*-th target as shown in Figure 1. In order to detect the range term, by multiplying the conjugation of $s^{\mathrm{TX}}\left(t\right)$, the dechirped signal $y_{k,l}\left(t\right)$ is obtained as follows:

(3) $$\begin{array}{cc} y_{k,l}\left(t\right) & =s_{k,l}^{\mathrm{RX}}\left(t\right)\times s^{*\mathrm{TX}}\left(t\right)\\ & =\sum_{m=1}^{M}{\dot{a}}_{m}\underset{\mathrm{range}\;\mathrm{term}}{\underset{︸}{\exp\left(j\pi \mu \tau_{m}t\right)}}\underset{\mathrm{Doppler}\;\mathrm{term}}{\underset{︸}{\exp\left(\frac{j4\pi lT}{\lambda }\left(x_{m}^{\left(\mathrm{hr}\right)}\left(t\right)+\sum_{h=1}^{H}x_{m,h}^{\left(\mathrm{rr}\right)}\left(t\right)\right)\right)}}\underset{\mathrm{DOA}\;\mathrm{term}}{\underset{︸}{\exp\left(\frac{j2\pi }{\lambda }dk\sin\theta_{m}\right)}}+w\left(t\right) \end{array}$$

where ${\dot{a}}_{m}$ is the coefficient excluding the range term, ${\dot{a}}_{m}={\tilde{a}}_{m}\exp\left(-j\left(2\pi f_{c}\tau_{m}-\mu \tau_{m}^{2}/2\right)\right)$ [20] and w(t) is the noise term, that is, $w\left(t\right)=\tilde{w}\left(t\right)s^{*\mathrm{TX}}\left(t\right)$. Dechirped signal $y_{k,l}\left(t\right)$ is converted from analog to digital with sampling interval $t_{s}$ for $0\leq n\leq N_{s}-1$, where $N_{s}$ is the number of samples, that is, $N_{s}=T/t_{s}$. Then, it is denoted by $y_{k,l}\left[n\right]$ and expressed as follows:

(4) $$\begin{array}{c} y_{k,l}\left[n\right]=\sum_{m=1}^{M}{\dot{a}}_{m}\underset{\overset{\Delta }{\;=}\eta_{m}\left[n\right]}{\underset{︸}{\exp\left(j\pi \mu \tau_{m}nt_{s}\right)}}\underset{\overset{\Delta }{\;=}v_{m}^{l}\left[n\right]}{\underset{︸}{\exp\left(\frac{j4\pi lT}{\lambda }\left(x_{m}^{\left(\mathrm{hr}\right)}\left[n\right]+\sum_{h=1}^{H}x_{m,h}^{\left(\mathrm{rr}\right)}\left[n\right]\right)\right)}}\underset{\overset{\Delta }{\;=}h_{m}^{k}}{\underset{︸}{\exp\left(\frac{j2\pi }{\lambda }dk\sin\theta_{m}\right)}}+w\left[n\right]. \end{array}$$

In this paper, meanwhile, we focus only on DOA estimation; thus, the other parameters, that is, range term $\eta_{m}\left[n\right]$ and Doppler term $v_{m}^{l}\left[n\right]$ were omitted. By only considering DOA term $h_{m}^{k}$, that is, $a_{m,l}\left[n\right]={\dot{a}}_{m}\eta_{m}\left[n\right]v_{m}^{l}\left[n\right]$, Equation (4) is newly expressed as follows:

(5) $$\begin{array}{c} y_{k,l}\left[n\right]=\sum_{m=1}^{M}a_{m,l}\left[n\right]h_{m}^{k}+w_{k,l}\left[n\right]. \end{array}$$

Assuming that DOA term $h_{m}^{k}$ is invariant for sample index *n*, Equation (5) is simply expressed as:

(6) $$\begin{array}{c} y_{k,l}=\sum_{m=1}^{M}a_{m,l}h_{m}^{k}+w_{k}. \end{array}$$

For effective representations, variables are denoted into vector and matrix forms as follows:

(7) $$\begin{array}{c} Y=HA+W, \end{array}$$

where Y is the K×L matrix for $y_{k,l}$, W is the K×L AWGN matrix, and H is the K×M steering matrix, that is, $H={\left[h_{1},h_{2},...,h_{M}\right]}^{T}$ where $h_{m}$ is steering vector of the *m*th target, that is, $h_{m}\;=\;{\left[1,h_{m},h_{m}^{2},...,h_{m}^{K-1}\right]}^{T}$, A is the M×L amplitude matrix of column vectors $a_{l}={\left[a_{1,l},a_{2,l},...,a_{M,l}\right]}^{T}$ for *l*th chirp and ${\left(\cdot \right)}^{T}$ is the transpose operator of the matrix. Therefore, H and A are expressed as:

(8) $$\begin{array}{c} H=\left[h_{1},h_{2},...,h_{M}\right]\\ \;=\left[\begin{array}{cccc} 1 & 1 & \cdots & 1\\ h_{1} & h_{2} & \cdots & h_{M}\\ \vdots & \vdots & \ddots & \vdots \\ h_{1}^{K-1} & h_{2}^{K-1} & \cdots & h_{M}^{K-1} \end{array}\right], \end{array}$$

(9) $$\begin{array}{c} A=\left[a_{1},a_{2},...,a_{L}\right]\\ \;=\left[\begin{array}{cccc} a_{1,1} & a_{1,2} & \cdots & a_{1,L}\\ a_{2,1} & a_{2,2} & \cdots & a_{2,L}\\ \vdots & \vdots & \ddots & \vdots \\ a_{M,1} & a_{M,2} & \cdots & a_{M,L} \end{array}\right]. \end{array}$$

### 2.2. FOV and Resolution in DOA Estimation

This section illustrates the FOV and resolution in DOA estimation. FOV and resolution are important factors in DOA estimation. The DOA resolution is denoted by $\theta_{\Delta }$, and it is calculated as [20,33]

(10) $$\begin{array}{c} \theta_{\Delta }\approx 0.886\frac{\lambda }{Kd\cos\theta }, \end{array}$$

where θ is the angular position. From Equation (10), it can be observed that angle resolution $\theta_{\Delta }$ decreased as *d* increased in denominator. A smaller $\theta_{\Delta }$ means more improved performance of angle resolution, because a smaller $\theta_{\Delta }$ can distinguish the smaller angle difference of adjacent targets. Meanwhile, the FOV according to *d* is denoted by $\theta_{\mathrm{FOV}}$, and it is calculated as follows [TI MIMO]:

(11) $$\begin{array}{c} \theta_{\mathrm{FOV}}=\sin^{-1}\left(\frac{\lambda }{2d}\right). \end{array}$$

**Figure 3:**
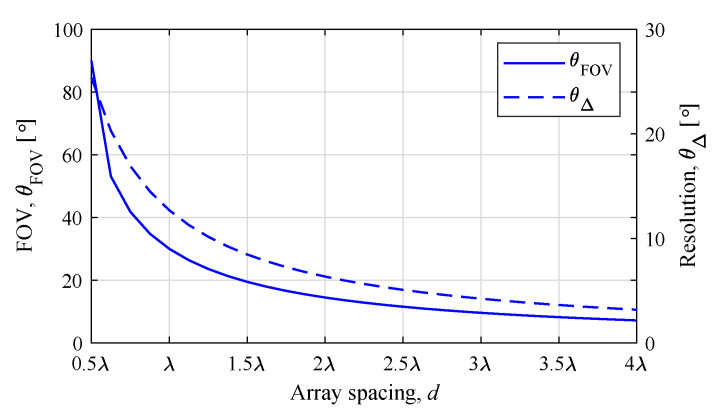
Field of view (FOV) and resolution according to array spacing *d* with *K* = 8.

Figure 3 shows $\theta_{\Delta }$ (dashed line) and $\theta_{\mathrm{FOV}}$ (solid line) according to *d* with K=8 using Equations (10) and (11), respectively. It can be seen that $\theta_{\Delta }$ decreased as array spacing *d* increased, as mentioned before. Meanwhile, $\theta_{\mathrm{FOV}}$ decreased as *d* increased.

**Figure 4:**
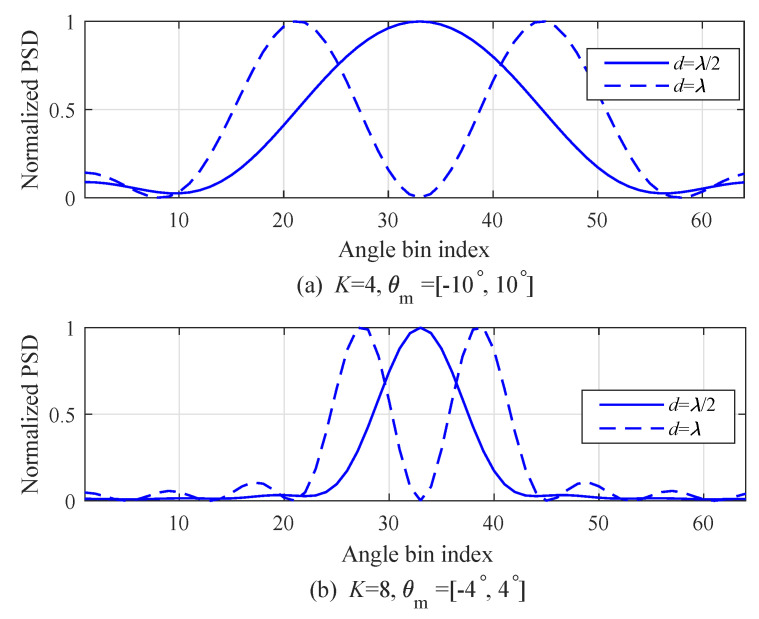
Normalized power spectral density (PSD) according to angle-bin index.

Figure 4 shows the normalized power spectral density (PSD) according to angle-bin index in order to illustrate the performance of angle resolution according to *d*. Figure 4a shows the result with the number of arrays K=4 and two DOA, that is, $\left[\theta_{1},\theta_{2}\right]=\left[-10^{{}^{\circ}},10^{{}^{\circ}}\right]$. In the case of d=λ/2 and $\theta =10^{{}^{\circ}}$, $\theta_{\Delta }$ is approximated into $25.77^{{}^{\circ}}$ by Equation (10) and $\theta_{\Delta }$ is larger than $|\theta_{2}-\theta_{1}|=20$. Hence, it was detected as if there was only one target DOA, as shown in the case of d=λ/2 in Figure 4a. On the other hand, in the case of d=λ, $\theta_{\Delta }$ was approximated into 12.89; thus, two angles were detected as shown in the case of d=λ in Figure 4a. In the same manner, Figure 4b shows the result with number of arrays K=8 and two DOA, that is, $\left[\theta_{1},\theta_{2}\right]=\left[-4^{{}^{\circ}},4^{{}^{\circ}}\right]$. As illustrated in Figure 4a, the estimation of two angles that was not possible with d=λ/2 was possible with d=λ.

**Figure 5:**
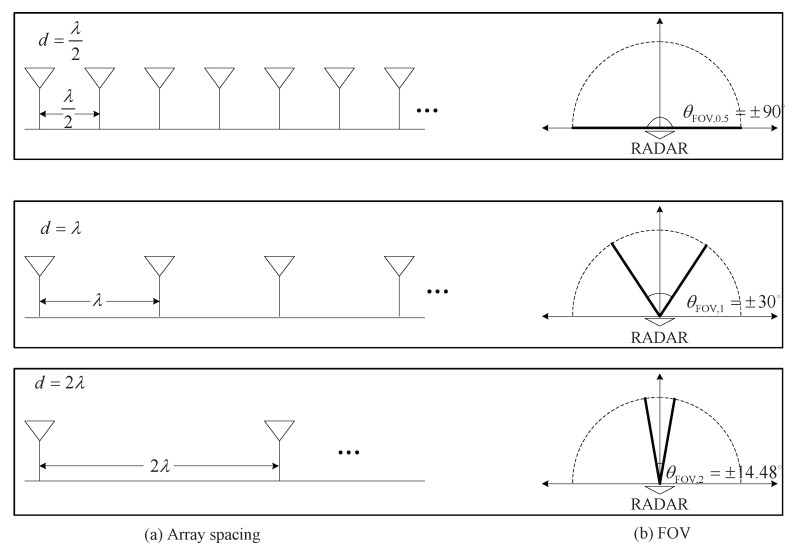
Schematic of relation between array spacing and FOV.

Figure 5 illustrates the schematic of the relation between array spacing and FOV. For easy denoting, $\theta_{\mathrm{FOV}}$ is expressed as $\theta_{\mathrm{FOV},0.5}$, $\theta_{\mathrm{FOV},1}$, and $\theta_{\mathrm{FOV},2}$ for d=λ/2, d=λ, and d=2λ, respectively, that is, $\theta_{\mathrm{FOV}}\in \left\{\theta_{\mathrm{FOV},0.5},\theta_{\mathrm{FOV},1},\theta_{\mathrm{FOV},2}\right\}$. In the case of d=λ/2, $\theta_{\mathrm{FOV}}$ is equal to $\pm \;90^{{}^{\circ}}$, that is, $\theta_{\mathrm{FOV},0.5}=\pm \;90^{{}^{\circ}}$. Meanwhile, in the cases of d=λ and d=2λ, $\theta_{\mathrm{FOV}}$ decreases to $\pm \;30^{{}^{\circ}}$ and $\pm \;14.48^{{}^{\circ}}$, respectively, that is, $\theta_{\mathrm{FOV},1}=\pm \;30^{{}^{\circ}}$ and $\theta_{\mathrm{FOV},2}=\pm \;14.48^{{}^{\circ}}$. The FOV decrease means that the angle of the detectable area in the radar system is significantly reduced. From these results, it can be concluded that there is a trade-off between resolution performance and FOV depending on array spacing *d*.

## 3. Frequency-Estimation Algorithms

### 3.1. FFT-Based Algorithm for DOA Detection

FFT-based spectral-estimation algorithms are the most widely used algorithm for various detections because of their many advantages, such as low complexity and ease of implementation. *N*-point FFT algorithms efficiently estimate the frequency components of input signals by calculating correlations with sinusoid signals of *N* frequencies. We recall Equation (6) in order to perform FFT for DOA detection. The *u*th FFT output for 0≤u≤N−1 is denoted by $Y_{u,l}$, and it is expressed as

(12) $$\begin{array}{c} Y_{u,l}=\sum_{k=0}^{N-1}y_{k,l}D_{N}^{ku}, \end{array}$$

where $D_{N}$ is the *N*-point DFT operator (So far, we mentioned FFT, but denote DFT instead of the FFT for mathematical expression), that is, $D_{N}=\exp\left(-j2\pi /N\right)$. For effective representations, Equation (12) is denoted into vector and matrix forms as follows:

(13) $$\begin{array}{c} Y_{\mathrm{DFT}}=DY, \end{array}$$

where D is the N×K matrix for a DFT operation that consisted of *K* column vectors, that is, $D=[D_{0},D_{1},...,D_{K}-1]$ where $D_{u}$ is the *u*th column vector of D, that is, $D_{u}\;=\;{\left[1,\;\;\exp\left(-j2\pi u/N\right),\exp\left(-j4\pi 2u/N\right),...,\exp\left(-j2\pi (N-1)u/N\right)\right]}^{T}$ for 0≤u≤K−1. Despite the merits of the low complexity of FFT, as shown in Figure 4, FFT may not estimate two angles in the case of $\theta_{\Delta }>\left|\theta_{2}-\theta_{1}\right|$. In order to estimate two angles despite $\theta_{\Delta }>\left|\theta_{2}-\theta_{1}\right|$, the super-resolution algorithms need to be mentioned in the next section.

### 3.2. MUSIC Algorithm

This section illustrates the MUSIC algorithm, which is a representative subspace-based spectral-estimation algorithm with super resolution [27,28,29]. The MUSIC algorithm employs the orthogonality between subspaces of signal and noise. In order to run the MUSIC algorithm, the correlation matrix of $y_{k}$ is employed, which is denoted by R and expressed as [27]:

(14) $$\begin{array}{c} \begin{array}{c} R=YY^{H}\\ \;\;\;=\left[\begin{array}{cccc} y_{0,0} & y_{0,1} & \cdots & y_{0,L-1}\\ y_{1,0} & y_{1,1} & \cdots & y_{1,L-1}\\ \vdots & \vdots & \ddots & \vdots \\ y_{K-1,0} & y_{K-1,1} & \cdots & y_{K-1,L-1} \end{array}\right]{\left[\begin{array}{cccc} y_{0,0} & y_{0,1} & \cdots & y_{0,L-1}\\ y_{1,0} & y_{1,1} & \cdots & y_{1,L-1}\\ \vdots & \vdots & \ddots & \vdots \\ y_{K-1,0} & y_{K-1,1} & \cdots & y_{K-1,L-1} \end{array}\right]}^{H}\\ \;\;\;=\left[\begin{array}{cccc} R_{0,0} & R_{0,1} & \cdots & R_{0,L-1}\\ R_{1,0} & R_{1,1} & \cdots & R_{1,L-1}\\ \vdots & \vdots & \ddots & \vdots \\ R_{K-1,0} & R_{K-1,1} & \cdots & R_{K-1,L-1} \end{array}\right]. \end{array} \end{array}$$

In order to perform the decomposition of the subspaces between signal and noise, singular decomposition (SVD) is employed as follows [29]:

(15) $$\begin{array}{c} R={U\Sigma U}^{H}, \end{array}$$

where Σ is a diagonal matrix based on *K* eigenvalues, that is, $\Sigma =diag(\lambda_{1},\lambda_{2},...,\lambda_{K})$ where $\lambda_{k}$ is the *k*th eigenvalue of R. *K* eigenvalues are given as

(16) $$\begin{array}{c} \lambda_{i}=\left\{\begin{array}{cc} \rho_{i}+\sigma_{n}^{2} & \mathrm{for}\;\;\;i=1,...,M\\ \sigma_{n}^{2} & \mathrm{for}\;\;\;i=M+1,...,K, \end{array}\right. \end{array}$$

where $\rho_{i}$ is the *i*th eigenvalue of the signal term, and $\sigma_{n}^{2}$ is the noise power. By separating the signal and noise terms, Equation (15) is expressed as follows:

(17) $$\begin{array}{c} R=U\Sigma U^{H}=\underset{\mathrm{signal}\;\mathrm{term}}{\underset{︸}{U_{M}\Sigma_{M}U_{M}^{H}}}+\underset{\mathrm{noise}\;\mathrm{term}}{\underset{︸}{\sigma_{n}^{2}U_{-M}}}, \end{array}$$

where $U_{M}$ corresponds to the signal term, that is, $U_{M}=\left[u_{1},u_{2},...,u_{M}\right]$ and $U_{-M}$ corresponds to the noise term, that is, $U_{-M}=\left[u_{M+1},u_{M+2},...,u_{K}\right]$. Then, the range of angle is uniformly partitioned within [−π/2,π/2] to a sample of $N_{\theta }$. By employing the orthogonality between signal and noise subspace matrices for $N_{\theta }$ candidates of the angle, the pseudospectrum denoted by $P_{\mathrm{MUSIC}}$ is calculated as

(18) $$\begin{array}{c} P_{\mathrm{MUSIC}}=\frac{1}{h^{H}\left(\theta \right)U_{-M}U_{-M}^{H}h\left(\theta \right)}. \end{array}$$

**Figure 6:**
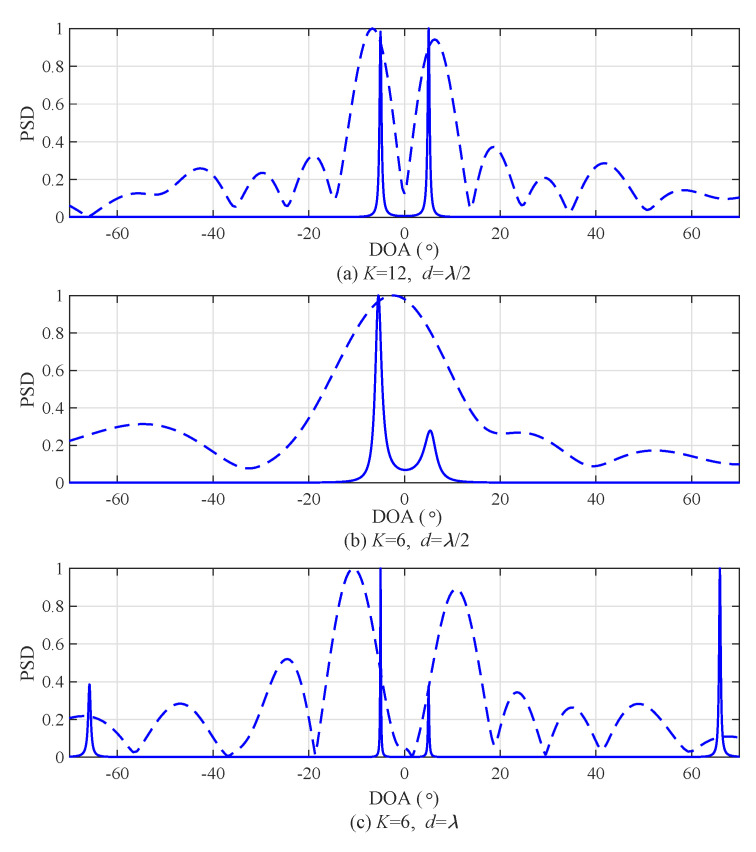
Power spectral density of multiple-single classifier (MUSIC) and fast Fourier transform (FFT) with d=λ/2 and d=λ.

Figure 6 shows the power spectral density (PSD) of the FFT and MUSIC algorithm with SNR = 10 dB and $\left[\theta_{1},\theta_{2}\right]=\left[-5^{{}^{\circ}},5^{{}^{\circ}}\right]$ in order to confirm the performance due to the distance between array *d* and number of arrays *K*. In Figure 6a, *K* was set to 12 and d=λ/2. In this case, both FFT and the MUSIC algorithm detected two angles because *K* was large enough to distinguish two angles. Meanwhile, in Figure 6b, *K* was reduced by half compared to the previous case, that is, K=6 and d=λ/2. As a result, the FFT was not able to distinguish the two angles. This implies that the FFT detected as if there was only one target when the number of arrays was insufficient despite there being two targets. On the other hand, the MUSIC algorithm distinguished the two angles due to characteristics of its super resolution. In Figure 6c, distance between adjacent arrays *d* was set to be twice that in Figure 6b, that is, K=6 and d=λ. As a result, in the case of FFT, the two angles were distinguished although the error of the angle-detection result slightly increased. In the case of the MUSIC algorithm, the estimation result became sharper compared to that in Figure 6b. Due to FOV reduction, there was ambiguity of the detection results at $-70^{{}^{\circ}}$ and $70^{{}^{\circ}}$.

## 4. Proposed Low-Complexity MUSIC Algorithm

**Figure 7:**
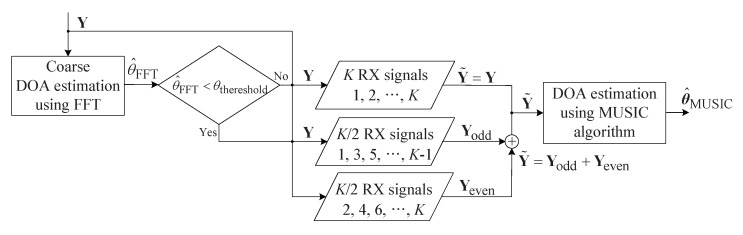
Structure of proposed algorithm.

This section describes the proposed low-complexity MUSIC algorithm based on coarse angle estimation by FFT. Figure 7 illustrates the structure of the proposed algorithm. As shown in Figure 7, the proposed algorithm performs the FFT on y in order to coarsely estimate the target angle. The estimated angle is denoted by ${\hat{\theta }}_{\mathrm{FFT}}$, and ${\hat{\theta }}_{\mathrm{FFT}}$ was compared to the threshold angle. The threshold angle is denoted by $\theta_{\mathrm{threshold}}$, and it was calculated as follows:

(19) $$\begin{array}{c} \theta_{\mathrm{threshold}}=\theta_{\mathrm{FOV},1}-\theta_{\Delta }\\ \;=30^{{}^{\circ}}-\theta_{\Delta }. \end{array}$$

**Figure 8:**
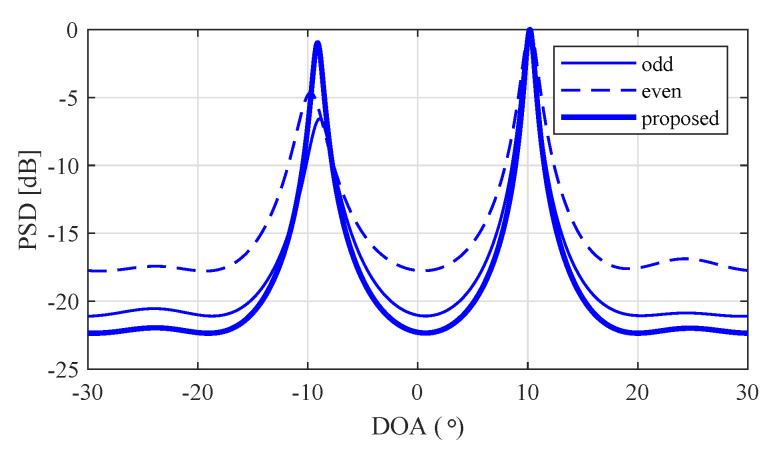
Snapshot of PSD of MUSIC algorithm according to input.

In the case of ${\hat{\theta }}_{\mathrm{FFT}}<\theta_{\mathrm{threshold}}$, since the target existed within $30^{{}^{\circ}}$, the proposed algorithm achieved similar performance, even when employing d=λ instead of d=λ/2. This implies that the MUSIC algorithm was run on only half the RX signal instead of the entire RX signal. The MUSIC input matrix is denoted by $\tilde{Y}$ and it was calculated as follows:

(20) $$\begin{array}{c} \tilde{Y}=Y_{\mathrm{odd}}+Y_{\mathrm{even}}, \end{array}$$

where $Y_{\mathrm{odd}}$ and $Y_{\mathrm{even}}$ are odd and even K/2 RX signals, respectively, that is, $Y_{\mathrm{odd}}$ is the composed of the 2k−1th row of Y for k=1,2,...,K/2 and $Y_{\mathrm{even}}$ is the composed of the 2kth row of Y for k=1,2,...,K/2. Then, in order to increase the signal-to-noise ratio (SNR), $Y_{\mathrm{odd}}$ and $Y_{\mathrm{even}}$ were summed. On the other hand, in the case of ${\hat{\theta }}_{\mathrm{FFT}}\geq \theta_{\mathrm{threshold}}$, the conventional MUSIC algorithm was run, that is, $\tilde{Y}$ was set to Y because *K* RX signals were employed as in the conventional MUSIC algorithm. Figure 8 shows a snapshot of the PSD of the MUSIC algorithm according to kinds of input in order to observe the SNR improvement of the proposed algorithm compared to odd and even signals.

In Figure 8, the distance between minimal and maximal values means the SNR. The minimal value of each PSD means noise power because the maximal value of each PSD was normalized to 1. From these results, it could be expected that the proposed algorithm achieved SNR improvement compared to odd and even signals.

**Figure 9:**
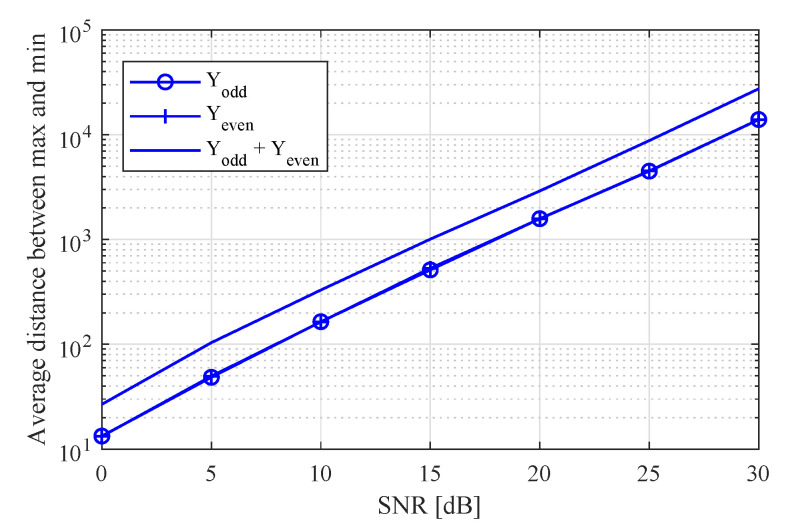
Distance between minimal and maximal values according to signal-to-noise ratio (SNR) with *K* = 6.

Figure 9 shows the average of the distance between the minimal and maximal values according to the SNR with K=6 in order to observe the SNR improvement of the proposed algorithm compared to the cases of $Y_{\mathrm{odd}}$ and $Y_{\mathrm{even}}$. In Figure 9, this implies that the SNR increased as the distance between minimal and maximal values was large. By normalizing the PSD with the maximal value, the peak value became 1, and the minimal value became the noise power. From these results, it can be observed that the proposed algorithm achieved a 3 dB SNR gain by simply merging the odd and even signals.

In conclusion, the difference between the proposed and the conventional MUSIC algorithms is as follows—first, in the proposed algorithm, it determines whether targets exist or not within the considered FOV range and reduces the number of inputs of the MUSIC algorithm. By doing so, the proposed algorithm reduces the overall complexity by eliminating unnecessary complexity while achieving performance similar to the conventional MUSIC algorithm. In the case when targets do not exist within the considered FOV, the proposed and the conventional MUSIC algorithms are the same. However, the complexity reduction effect by the proposed algorithm is drastic since the FOV is set narrow in the vital radar applications and it will be shown by simulations in the Section 5.

## 5. Simulation Results

This section introduces the simulation results in order to confirm the performance improvement of the proposed algorithm. First, we illustrate the performance evaluation. Then, we compare the computational complexity between the conventional and the proposed MUSIC algorithms.

### 5.1. Performance Evaluation

In order to compare the performance evaluation of each algorithm, the root-mean-square error (RMSE) of DOA estimation was employed. Simulations were performed for a total $10^{5}$ times. For all simulations, the center frequency was set to 24 GHz, and the complex amplitude was independently and randomly generated from uniform distribution. The RMSE was calculated as follows [20]:

(21) $$\begin{array}{c} \mathrm{RMSE}=\sqrt{\frac{1}{M\times 10^{5}}\sum_{i=1}^{10^{5}}\sum_{m=1}^{M}{\left(\theta_{m}-{\hat{\theta }}_{m}\right)}^{2}}. \end{array}$$

**Figure 10:**
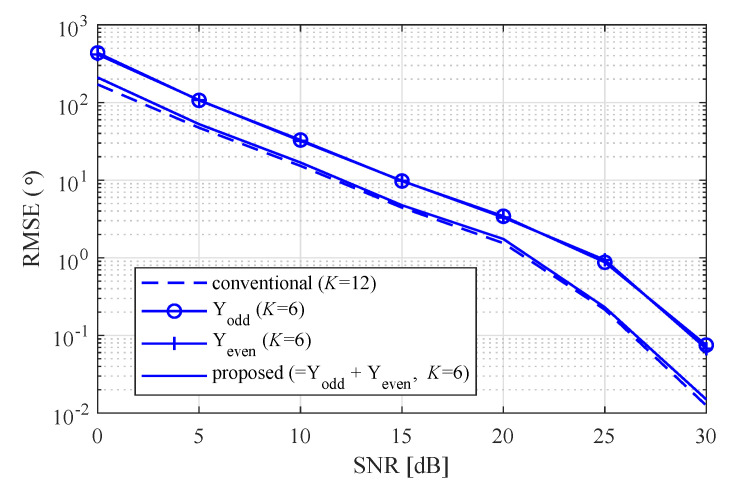
Root-mean-square error (RMSE) according to SNR with *K* = 12 and 6.

Figure 10 shows the RMSE according to the SNR with *K* = 12 and 6 and $\left[\theta_{1},\theta_{2}=-9^{{}^{\circ}},10^{{}^{\circ}}\right]$. From Figure 10, it can be seen that the proposed algorithm achieved almost the same performance as that of the conventional MUSIC algorithm, even though only half of the input was used compared to the conventional algorithm. Meanwhile, it was confirmed that proposed algorithm achieved 3 dB SNR improvement compared to cases using the same number of inputs, that is, $Y_{\mathrm{odd}}$ and $Y_{\mathrm{even}}$.

### 5.2. Complexity Comparison

This section evaluates the computational complexity of the conventional and the proposed MUSIC algorithms. In order to measure complexity, the required number of multiplications for the main operations for MUSIC algorithm were employed. Table 1 shows the required number of multiplication for the main operations for MUSIC algorithm, such as EVD and the generation of noise subspace.

In the case of the conventional MUSIC algorithm, angle detections are performed regardless of target FOV. Hence, the required number of multiplications of conventional MUSIC algorithm $C_{\mathrm{conventional}}$ is as follows:

(22) $$\begin{array}{c} C_{\mathrm{conventional}}=\frac{LK(K+1)}{2}+\frac{16}{5}K^{3}+\frac{K(K-M)(K+1)}{2}. \end{array}$$

On the other hand, the proposed algorithm performed FFT in order to determine whether the angle of target is smaller than $\theta_{\mathrm{threshold}}$. Then, in the case of ${\hat{\theta }}_{\mathrm{FFT}}\geq \theta_{\mathrm{threshold}}$, *K* is reduced to K/2. Otherwise, the complexity of the proposed algorithm is the same as that of the conventional MUSIC algorithm. Hence, the required number of multiplications of proposed algorithm $C_{\mathrm{proposed}}$ is as follows:

(23) $$\begin{array}{c} C_{\mathrm{proposed}}=\frac{N_{A}}{2}\log_{2}N_{A}+\left(1-p_{r}\right)C_{\mathrm{conventional}}+p_{r}\left(\frac{LK(K+2)}{8}+\frac{2}{5}K^{3}+\frac{K(K+2)(K-2M)}{8}\right)\\ \;=\frac{N_{A}}{2}\log_{2}N_{A}+C_{\mathrm{conventional}}-\frac{p_{r}}{40}K\left(10K+10L+127K^{2}+15KL-10KM\right), \end{array}$$

where $p_{r}$ is the probability that a target exists within $\theta_{\mathrm{threshold}}$, and $N_{A}$ is the FFT size for angle detection.

**Figure 11:**
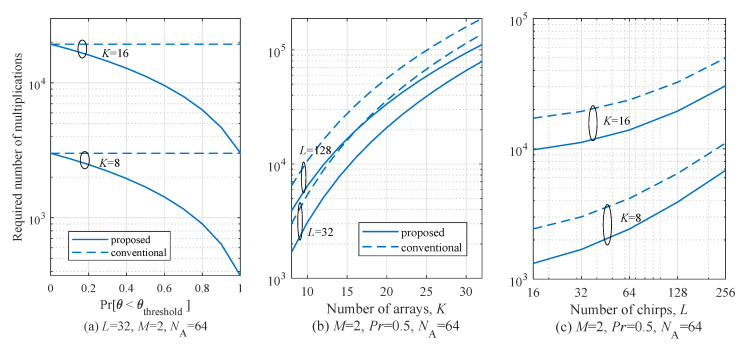
Comparison of complexity between conventional and proposed MUSIC algorithms for several parameters.

Figure 11 shows the required number of multiplications for several conditions. Various parameters were set to check the effect of each parameter on the complexity. Figure 11a shows the required number of multiplications according to $p_{r}$. As shown in Figure 11a, the number of arrays *K* is considered to 4 and 8. The total number of chirp symbols *L*, the number of targets *M*, and the size of the FFT for angle estimation $N_{A}$ were set to 32, 2, and 64, respectively. In the case of $p_{r}=0$, the complexity of the conventional and proposed algorithms was almost the same. On the other hand, the computational complexity of the proposed algorithm drastically decreased as $p_{r}$ increased to 1. Especially in the case of $p_{r}=0.5$, it can be seen that complexity was reduced by almost half compared to that of the conventional MUSIC algorithm. In the case of $p_{r}=1$, the proposed algorithm achieved six times lower complexity than that of the conventional MUSIC algorithms. Figure 11b shows the required number of multiplications according to number of arrays *K*. The parameters for simulations, *L* is set to 32 and 128 and $M,p_{r}$, and $N_{A}$ were set to 2, 0.5, and 64, respectively. From Figure 11b, it can be observed that the proposed algorithm achieved 42.4% complexity reduction on average compared to the conventional MUSIC algorithm. Similarly, Figure 11c shows the required number of multiplications according to number of chirps *L*. The parameters for simulations, $K,M,p_{r}$, and $N_{A}$ were set to 12, 2, 0.5, and 64, respectively. In this case, the proposed algorithm achieved 41.0% complexity reduction on average compared to that of the conventional MUSIC algorithm. In conclusion, from these results, it can be observed that as *L* and *K* increase, the complexity also increases but *L* and *K* do not affect the tendency of increase of complexity. On the other hand, the $p_{r}$ is the dominant parameter in terms of increase of the complexity. Meanwhile, the vital monitoring is performed near the radar, and the $p_{r}$ is almost 1 because the beam width of the considered radar system is 26 degrees [20,35]. Therefore, it is expected that the complexity reduction effect by the proposed algorithm is significant.

## 6. Experiments

In order to confirm the effectiveness of the proposed algorithm in a practical environment, we performed experiments inside an anechoic chamber, located at DGIST in Korea. This section consists of two subsections. First, we introduce the experiment equipment and then illustrate the experiment results.

### 6.1. Experiment Setup

**Figure 12:**
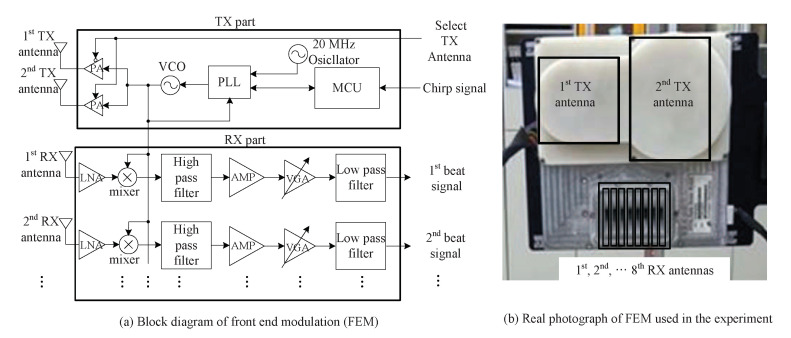
Block diagram of front-end module (FEM) and its real photograph.

For the experiment, we employed an FMCW radar system with $f_{c}=$ 24 GHz that had two TX antennas and eight RX antennas, as shown in Reference [20]. Figure 12 shows a block diagram of 24 GHz FMCW radar employed in experiment. The front-end module (FEM) was composed of TX and RX parts, as shown in Figure 12a,b. A microcontroller unit (MCU), frequency synthesizer with a phase-locked loop (PLL), and a voltage-controlled oscillator (VCO) were included on the TX side, with a maximal bandwidth of 2 GHz. The MCU chip controlled the frequency synthesizer with the PLL. Lastly, the output of the VCO was connected to the two TX antennas through a power amplifier (PA). RX signals through the eight antennas were improved and mixed by a low-noise-amplifier (LNA) mixer. Then, the beat signals were obtained through a high-pass filter, amplifier (AMP), variable gain amplifier (VGA), and low-pass filter.

**Figure 13:**
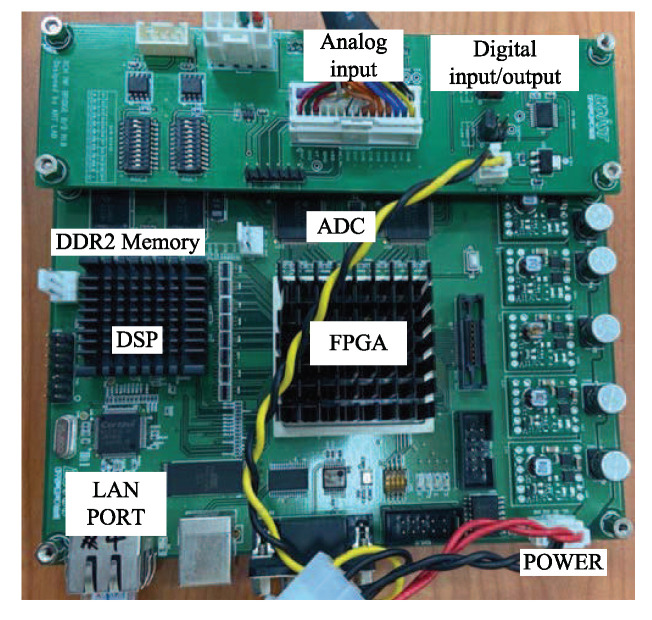
Back-end module (BEM).

Figure 13 shows the back-end-module (BEM) system used in the experiment [20]. As shown in Figure 13, the BEM included a field programmable gate array (FPGA) and digital-signal processing (DSP). The two 2 GB DDR2 SDRAMs were external memories of the DSP, providing a total of 512 Mbytes of data-storage space. The analog signal was converted to digital data with 20 MHz sampling rate through the ADC. The external memory was filled; then, data were transferred to the computer through the local-area-network (LAN) port.

**Figure 14:**
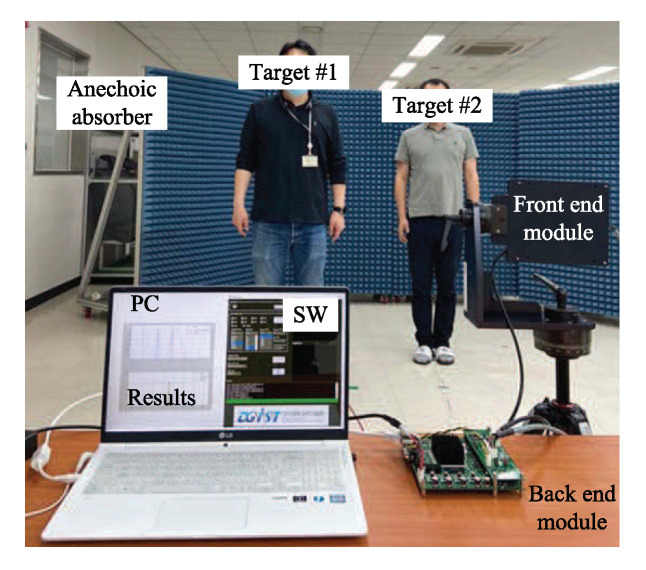
Experiment environment.

Figure 14 shows the experiment environment. As shown in Figure 14, two persons were positioned and stopped at the same and different distances from the radar. The reason for considering the stop targets is that the Doppler due to movement of target is dominant than the Doppler caused by the respiration signal. In order to reduce negative effects due to undesired reflected signals, an anechoic absorber was installed behind the targets. The duration of the chirp (ramp) *T* was set to 400 μs, bandwidth *B* was set to 1 GHz, and sampling frequency $f_{s}$ is set to 5 MHz. The number of chirps per one frame *L* was set to 256, the number of targets *M* was set to 2, the number of arrays *K* was set to 8, and the number of total frames was set to 64. For DOA estimation, $N_{A}$ is set to 64. Therefore, the range resolution is determined to 0.15 m, that is, range resolution is calculated as ΔR=c/2B where *c* is the velocity of radiation. For the range estimation, the size of FFT is denoted by $N_{R}$ and it is set to 2048 and thus the size of one range bin is calculated as 0.1465 m, that is, $\Delta R_{b}=cTf_{s}/2BN_{R}$ = 0.1465. The Doppler resolution ΔD is calculated as 3.6 b/m, that is, $\Delta D=60c/2LTf_{c}$. The range and Doppler resolutions affect the error margin of the result of parameter estimation.Through FEM, the RF signal was transmitted and received. Through the BEM, the ADC signal was obtained and restored. On a personal computer (PC), the conventional and proposed algorithms employed the restored signal from the BEM.

### 6.2. Experiment Results

**Figure 15:**
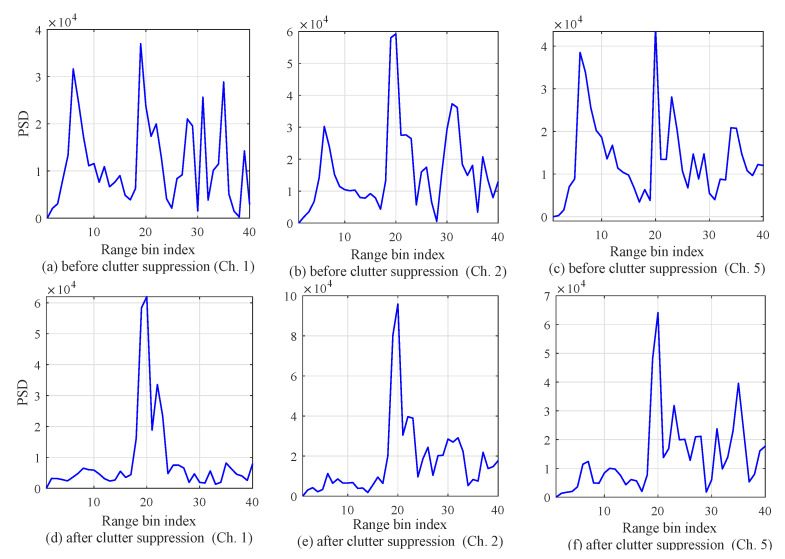
Experiment results of range detection from multiple channels. (**a**) Before clutter suppression with Ch. 1; (**b**) after clutter suppression (Ch. 1); (**c**) before clutter suppression (Ch. 2); (**d**) after clutter suppression (Ch. 2); (**e**) before clutter suppression (Ch. 5); (**f**) after clutter suppression (Ch. 5).

This section addresses the experiment results to confirm the improvement by the proposed algorithm. Figure 15 shows the experiment results of range detection from multiple channels. Figure 15a–c shows the PSD according to the range-bin index before clutter suppression. On the other hand, Figure 15d–f shows the PSD according to the range-bin index after clutter suppression. From these figures, it can be observed that the severe clutter terms were removed. Figure 15d–f shows that the PSD peak was detected at the 20th range-bin index. On the basis of this index, the range was estimated.

**Figure 16:**
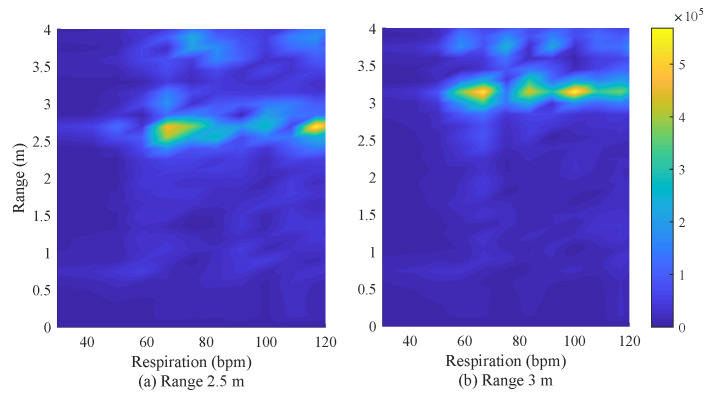
Experiment results of respiration rate and range detection.

Figure 16 shows the experiment results of respiration rate and range detection for two persons. The focus of this paper is the estimation of DOA of targets but these results are shown as an intermediate process for DOA estimation. As shown in Figure 14, two persons are considered and they were set to be located at the same range. Figure 16a shows two persons at a distance of 2.5 m from radar and Figure 16b shows a case at 3 m. In Figure 16a, it can be observed that two targets corresponding to 60 and 110 b/m of respiration were detected at the 2.5 m region. In the same manner, in Figure 16b, it can be seen that two targets corresponding to 60 and 100 b/m of respiration were detected at the 3 m region.

**Figure 17:**
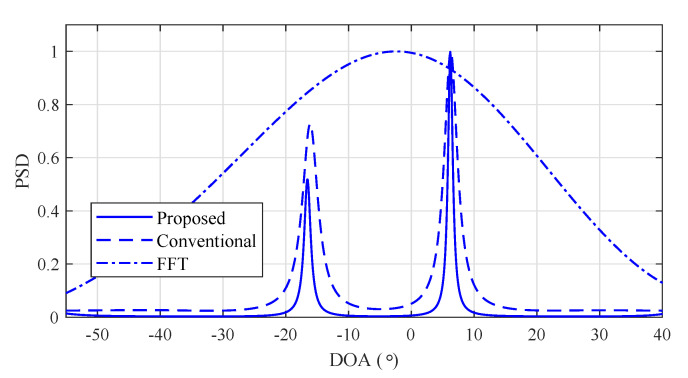
Experimental results of FFT, conventional, and proposed MUSIC algorithms.

Figure 17 shows the experiment results of FFT, and the conventional and the proposed MUSIC algorithms. From the FFT result, it can be seen that FFT was not able to estimate two targets due to its lack of resolution. On the other hand, the conventional and the proposed MUSIC algorithms could distinguish two adjacent targets. It can especially be observed that the proposed algorithm achieved the estimation of two adjacent targets as in the conventional algorithm, although the complexity of the proposed algorithm was significantly reduced.

## 7. Conclusions

We proposed a low-complexity MUSIC-based DOA detection algorithm for an FMCW vital radar. The proposed algorithm employed the characteristics that the distance between adjacent arrays has trade-offs between FOV and resolution performance. The proposed algorithm reduced computational complexity by decreasing the number of inputs of the MUSIC algorithm when the estimated DOA was within 30${}^{{}^{\circ}}$. As the simulation and experiment results showed, the proposed algorithm achieved similar performance to that of the conventional MUSIC algorithm while reducing computational complexity.

## Figures and Tables

**Table 1:** Required number of multiplications of main operations for MUSIC algorithm [34].

Operation	Equation	Required Number of Multiplications
Generation of covariance matrix R	$YY^{H}$	$\frac{LK(K+1)}{2}$
SVD calculation	$U\Sigma U^{H}$	$\frac{16}{5}K^{3}$
Noise-subspace generation	$U_{-M}U_{-M}^{H}$	$\frac{K(K+1)(K-M)}{2}$
Pseudospectrum calculation $P_{\mathrm{MUSIC}}$	$\frac{1}{h^{H}\left(\theta \right)U_{-M}U_{-M}^{H}h\left(\theta \right)}$	$N_{\theta }K\left(K+1\right)$
